# Phenotypic Expansion and Molecular Implications in Recessive *FUZ* ‐Related Ciliopathy

**DOI:** 10.1111/cge.70170

**Published:** 2026-04-08

**Authors:** Yosuke Ogawa, Shota Kato, Kazuhiro Shiraga, Motohiro Kato, Ryo Inuzuka

**Affiliations:** ^1^ Department of Pediatrics The University of Tokyo Hospital Tokyo Japan; ^2^ Department of Pediatrics, Graduate Department of Pediatrics, Graduate Department of Pediatrics, Graduate School of Medicine The University of Tokyo Tokyo Japan; ^3^ Department of Genome Informatics, Graduate Department of Genome Informatics, Graduate Department of Genome Informatics, Graduate School of Medicine The University of Tokyo Tokyo Japan

**Keywords:** aorto‐pulmonary window, ciliopathy, *FUZ*, primary cilia, sonic hedgehog

## Abstract

*FUZ*, a component of the CPLANE (ciliogenesis and planar polarity effector) complex, regulates primary ciliogenesis. Five patients of various types of skeletal dysplasia with biallelic *FUZ* variants have been reported to date, yet the gene‐disease relationship has not been established. Here, we report a patient with ciliopathy with a novel homozygous missense variant in *FUZ*. This patient shares phenotypes with all the previously reported patients and presents with novel phenotypes: aorto‐pulmonary window (AP window) and Hirschsprung disease. These phenotypes can be explained by the inhibition of neural crest cell migration due to abnormal Sonic hedgehog (Shh) signaling caused by primary cilia dysfunction. In silico three‐dimensional structural analysis predicted that the variant alters interactions between *FUZ* and *CPLANE2* (*RSG1*), potentially disrupting ciliogenesis. This report provides additional evidence for *FUZ* as a causative gene for ciliopathy, offering novel insights into the phenotype spectrum and molecular mechanisms of *FUZ*‐related ciliopathy.

## Graphical abstract

Our patient with homozygous *FUZ* p.Arg234Trp, potentially altering FUZ‐CPLANE2 interactions, presented with aorto‐pulmonary window, Hirschsprung disease, and shared phenotypes with previously reported ciliopathy patients. This report provides additional evidence for *FUZ* as a causative gene for ciliopathy, offering novel insights into the phenotype spectrum and molecular mechanisms of *FUZ*‐related ciliopathy.

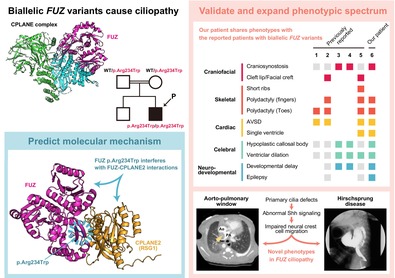

## 1 Introduction

Ciliopathy is a defect in primary cilia that causes developmental abnormalities in multiple organs. Numerous genes are involved in primary cilia development and function [1]. *FUZ (fuzzy planar cell polarity protein)*, along with *WDPCP (WD repeat‐containing planar cell polarity effector)* and *INTU (inturned planar cell polarity protein)*, form the CPLANE (ciliogenesis and planar polarity effector) complex functioning in primary ciliogenesis and intraflagellar transport [2, 3, 4].

Recently, five patients across four families with biallelic pathogenic variants in *FUZ* have been reported, presenting with a wide variety of skeletal dysplasia, including short rib polydactyly syndrome, craniosynostosis, and orofacial‐digital syndrome [5, 6, 7]. Homozygous *FUZ* mutant or knock‐out mice also exhibit skeletal ciliopathy phenotypes [2, 3]. However, the gene‐disease relationship remains unestablished in Clinical Genome Resource (ClinGen), the full multi‐organ phenotypic spectrum is incompletely characterized, and genotype–phenotype correlations remain unclear. These knowledge gaps limit the clinical utility of genetic findings in *FUZ* for definitive diagnosis, comprehensive patient evaluation, and prognostic counseling.

Recent structural and functional studies have elucidated the molecular basis of *FUZ*‐related ciliogenesis. WDPCP, INTU, and FUZ form a crescent‐shaped CPLANE complex that interacts with the small GTPase CPLANE2 (RSG1), which is essential for primary ciliogenesis [4, 8, 9]. Understanding this complex architecture and interaction with CPLANE2 has provided a framework for interpreting pathogenic variants. Advanced structural prediction tools [10] further enable assessment of how specific variants disrupt these molecular interactions.

We report a patient with an extremely rare homozygous *FUZ* variant presenting with characteristic skeletal phenotypes and two previously unreported manifestations: aorto‐pulmonary window (AP window) and Hirschsprung disease. Through clinical, genetic, and structural analyses, we provide additional evidence for *FUZ* as a causative gene for ciliopathy and offer insights into its molecular mechanisms and phenotypic spectrum.

## 2 Materials and Methods

We collected the clinical course and imaging findings from the patient. Whole‐exome sequencing of the patient was performed with family consent. The library was constructed using the SureSelect V6 kit and sequenced on the NovaSeq 6000 system (Illumina, CA, USA). Sequence data were mapped using BWA MEM, jointly called using GATK best practice, and annotated via Ensembl VEP. Obtained variants were filtered based on allele frequency, in silico pathogenicity predictions, and gene function (Table S1). The final candidate *FUZ* variant was confirmed by Sanger sequencing in the patient and parents. We visualized the structure of the human CPLANE complex (PDB ID: 7Q3D) with PyMOL and utilized the APBS plugin to display molecular surface charge. The interactions between human FUZ and CPLANE2 molecules were predicted using AlphaFold3 [10].

## 3 Results

### 3.1 Clinical Presentation

**FIGURE 1:**
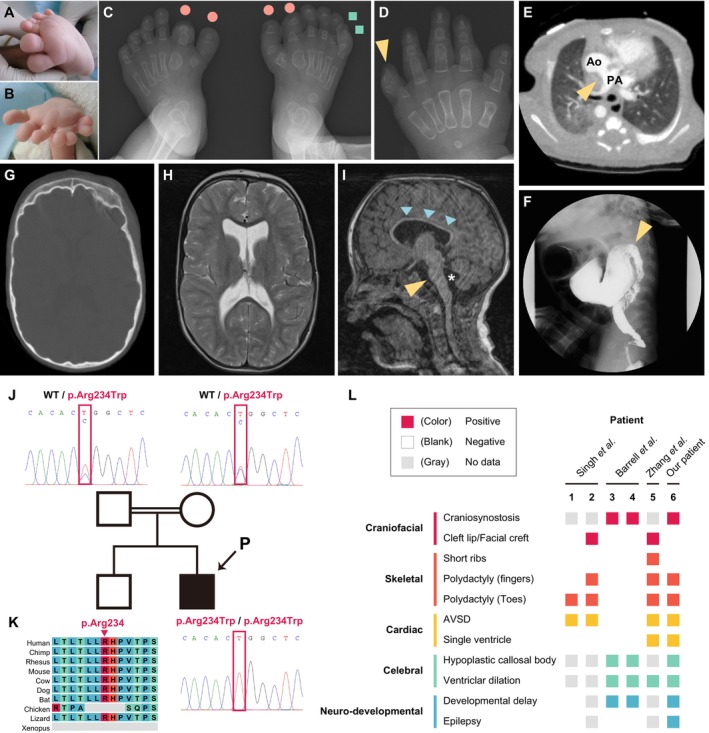
Genetic and clinical findings. A–C. Preaxial polydactyly (orange circles) and postaxial polysyndactyly (green squares) of the foot.D. Polysyndactyly of the left hand (yellow arrowhead).E. Contrast‐enhanced CT scan revealed aorto‐pulmonary window (yellow arrowhead). F. Lower gastrointestinal series showed a calibre change in the splenic flexure (yellow arrowhead) and a narrow segment from the descending colon to the sigmoid colon. G. Head CT at 5 years of age showed a narrow elongated cranium with indentations, consistent with sagittal craniosynostosis. H. Brain MRI horizontal T2‐weighted imaging at 4 years showed mild dilation of the lateral ventricles and decreased volume of the deep cerebral white matter. I. Brain MRI sagittal FLAIR imaging at 4 years showed hypoplasia of the callosal body (blue arrowheads) and pons (yellow arrowhead) and dilation of the fourth ventricle (white asterisk). J. Family pedigree and Sanger sequencing. The double line in the family pedigree indicates self‐reported possible consanguinity of undetermined degree. K. Amino acid sequence alignment of FUZ orthologs across different species. L. Graphical list of major phenotypes observed in the previously reported six patients with recessive FUZ‐related ciliopathy, including our patient. Colored squares indicate the presence of the phenotype, and gray squares indicate no data available.

The Japanese male patient, prenatally diagnosed with cardiac anomalies and limb shortening, was born at 36 weeks 6 days of gestation with birth weight 1943 g (−2.3 SD) and body length 42.0 cm (−2.1 SD). He had no family history, and his parents self‐reported possible consanguinity, although this could not be confirmed. Physical examination at birth revealed micrognathia, high‐arched palate, and polydactyly (Figure 1A–D). Chest X‐ray showed a normal thorax. Cardiac echocardiography and contrast‐enhanced CT revealed a complete atrioventricular septal defect (AVSD), double outlet right ventricle (DORV), and AP window (Figure 1E). He also presented with abdominal distension and vomiting since birth and was diagnosed with Hirschsprung disease by lower gastrointestinal series and rectal biopsy (Figure 1F). The patient underwent a Glenn operation and AP window repair at 9 months and a Hirschsprung disease operation at 1 year of age. In his infancy, developmental delay and epilepsy became apparent. Head CT and MRI at age 4 revealed scaphocephaly and multiple cerebral malformations, including: (1) hypoplasia of the pons, middle cerebellar peduncle, and cerebellar vermis; (2) enlargement of the fourth ventricle; (3) thin corpus callosum; and (4) reduced volume of deep cerebral white matter (Figure 1G–I). He is currently 12 years old and has severe intellectual disability and autism spectrum disorder.

### 3.2 Genetic Analysis

Whole‐exome sequencing identified an extremely rare homozygous missense variant in *FUZ*, p.Arg234Trp. Sanger sequencing confirmed that both asymptomatic parents were heterozygous for this variant (Figure 1J). The allele frequency of this variant is 0.000117 (14 heterozygotes) in the Japanese database (ToMMo; 60KJPN), and 0.0000077 (12 heterozygotes) in the gnomAD database v4.1. This variant has not been reported as homozygous in the general population, and its allele frequency is comparably rare to those of the reported pathogenic variants in recessive *FUZ*‐related ciliopathy (Table 1). *In silico* pathogenicity assessment showed deleterious effects in SIFT and PolyPhen, with a CADD score of 27.3 and an AlphaMissense score of 0.844 (likely pathogenic). This variant is highly conserved across a wide range of mammalian species (Figure 1K) and is located within a domain (LD2) that plays a key role in primary ciliogenesis [4].

**TABLE 1:** List of reported variants in recessive *FUZ*‐related ciliopathy.

Report	Variant	Type	AF_gnomAD	AFmax_gnomAD	Hom_gnomAD	SIFT	PolyPhen	CADD	AlphaMissense
Zhang et al. [7]	c.98_111+9del	Frameshift	0.000015	0.00031 (AFR)	0	—	—	26.3	—
Singh et al. [5]	p.Glu201Lys	Missense	0.0000064	0.00010 (SAS)	0	0.008	0.236	24.0	0.9016
Singh et al. [5]	p.Val209_Leu212del	In‐frame deletion	—	—	—	—	—	—	—
Our patient	p.Arg234Trp	Missense	0.0000077	0.00014 (EAS)	0	0	0.999	27.3	0.8440
Barrell et al. [6]	p.Arg284Pro	Missense	0.0000019	0.00017 (MID)	0	0.009	0.995	25.4	0.2714

Abbreviations: AF_gnomAD, allele frequency in gnomAD v4.1; AFmax_gnomAD, allele frequency in the genetic ancestry group with the highest allele frequency in gnomAD v4.1; AFR, African; EAS, East Asian; Hom_gnomAD, number of individuals homozygous in gnomAD v4.1; MID, Middle Eastern; SAS, South Asian.

### 3.3 In Silico Structural Analysis

**FIGURE 2:**
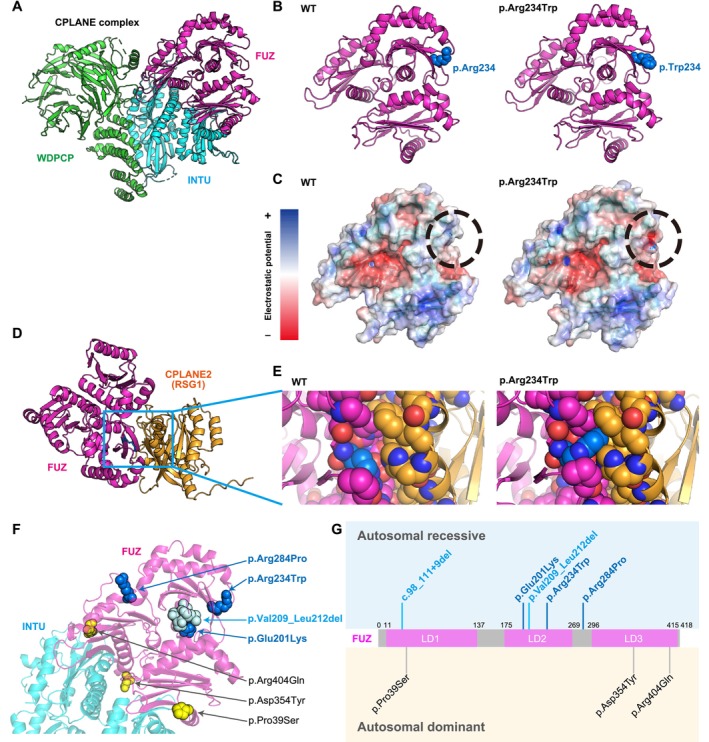
In silico structural analysis of FUZ. A. Cartoon representation of human CPLANE complex (PDB ID: 7Q3D), consisting of WDPCP, INTU, and FUZ. B. Wild type and p.Arg234Trp of FUZ, showing that the variant (indicated by blue molecule) is opposite to INTU. C. Electrostatic potential map of FUZ wild type and p.Arg234Trp, showing that the variant changes the electrostatic potential in the black dashed circle. D. FUZ and CPLANE2 (RSG1) interaction predicted by AlphaFold3. WDPCP and INTU are omitted and FUZ is vertically inverted from Figure 2A to make it easier to see the interaction. E. Enlarged view of the contact surface between FUZ and CPLANE2. Amino acids located at the contact surface are shown as molecular models. The p.Arg234Trp molecule (blue) extends toward CPLANE2 compared to the wild type. F. FUZ model with reported variants of autosomal recessive ciliopathy (blue or light blue molecules) and autosomal dominant neural tube defects (yellow molecules). G. Gene map of FUZ with three longin‐like domains (LD) and reported variants of autosomal recessive ciliopathy (upper) and autosomal dominant neural tube defects (lower).

FUZ, INTU, and WDPCP form the crescent‐shaped CPLANE complex (Figure 2A). The variant in this patient is located away from the surface where FUZ interacts with WDPCP and INTU (Figure 2B), and the surrounding electrostatic potential becomes negative due to the variant (Figure 2C). FUZ in the CPLANE complex also interacts with CPLANE2 (RSG1), regulating primary ciliogenesis [4, 8, 9]. At the AlphaFold3‐predicted interaction surface between FUZ and CPLANE2 (Figure 2D), a tryptophan residue of the variant protruded toward the CPLANE2 side, potentially interfering with their interaction (Figure 2E).

To date, six patients with recessive *FUZ*‐related skeletal ciliopathy, including our patient, have been reported [5, 6, 7] (Figure 1L **and** Table S2). Additionally, three patients with neural tube defects caused by dominant *FUZ* mutations have been reported [11]. The three dominant variants are located within the longin‐like domain (LD) 1 and LD3, which are on the surface adjacent to the INTU‐binding region. In contrast, most of the six recessive variants (excluding deletions) are located within the LD2, distant from the INTU‐binding surface (Figure 2F,G).

## 4 Discussion

We reported a 12‐year‐old male patient with an extremely rare homozygous missense variant in *FUZ* who presented with AP window and Hirschsprung disease, as well as overlapping phenotypes to previously reported patients. This variant is classified as likely pathogenic in the ACMG/AMP guidelines: PM1 (located in a functionally important domain where reported pathogenic variants are concentrated), PM2 (absent as homozygous in general databases), PP3 (predicted to be deleterious in multiple *in silico* tools), and PP4 (combination of clinical phenotypes specific for recessive *FUZ*‐related ciliopathy). *In silico* structural analysis predicted that the variant causes steric hindrance at the interaction surface with CPLANE2.

This patient shares multi‐organ phenotypes with those of each of the reported patients (Figure 1L **and** Table S2). The overlapping clinical features across patients, including craniofacial abnormalities, skeletal dysplasia, cardiac defects, and neurological manifestations, are consistent with a unified disease entity despite phenotypic heterogeneity. These phenotypes have also been reproduced in model mice. Homozygous *FUZ* knockout mice were repeatedly reported to exhibit craniofacial malformations (high‐arched palate [3, 12], cleft palate [13], and micrognathia [2, 12, 13]), skeletal abnormalities (polydactyly of the limbs [2, 3, 11], malformed ribs, and long bones [2, 13]), cardiac defects (conotruncal defects [2]), and cerebral malformations (ventricular enlargement [2] and hypoplastic hindbrain [14]); these phenotypes closely resemble those observed in patients with recessive *FUZ*‐related ciliopathy.

Notably, this patient presented with two previously unreported phenotypes in *FUZ*‐related ciliopathy: AP window and Hirschsprung disease. Both can be explained through Sonic hedgehog (Shh) signaling disruption affecting cardiac development. In ciliopathies, Shh abnormalities cause septal defects such as AVSD through impaired mesenchymal cell migration [15], and outflow tract defects through neural crest cell dysfunction [16, 17]. AP window, a rare outflow tract malformation with no prior ciliopathy association, likely arises through the latter neural crest‐mediated mechanism. The same pathway explains Hirschsprung disease: impaired neural crest cell migration prevents colonization of the developing intestine, resulting in absent enteric ganglia. Hirschsprung disease with the same neural crest migration defect has been reported in other ciliopathies, including Bardet‐Biedl syndrome [18], supporting the role of this pathway in ciliopathy‐associated Hirschsprung disease. Although we have not been able to validate these phenotypes through functional analysis, a single outflow tract, a conotruncal phenotype analogous to AP window, has been observed in homozygous *FUZ* knockout mice [2]. Hirschsprung disease has not been reported in *FUZ* animal models to date, possibly because previous reports on mouse models have focused on craniofacial, skeletal, and neurological phenotypes without systematically evaluating thoracic and abdominal organs [2, 3, 11, 12, 13, 14]; future research in this area may serve to validate this novel phenotype. The diverse phenotypes observed in this patient (spanning skeletal, cardiac, and gastrointestinal systems) can be unified through a common pathogenic mechanism of primary cilia dysfunction disrupting Shh‐mediated neural crest development, suggesting the pleiotropic effects of *FUZ* variants.

Structural analysis suggests that the *FUZ* variant in our patient is located on the FUZ‐CPLANE2 interaction surface, suggesting it may impair this functional interaction rather than disrupting CPLANE complex assembly. This finding aligns with a broader pattern: recessive ciliopathy variants tend to localize to the LD2 domain (away from the INTU interface) [5, 6, 7], while dominant neural tube defect variants cluster in LD1/LD3 domains (near the INTU interface) [11]. We hypothesize that variant position may influence both the molecular mechanism and phenotypic spectrum. LD2 variants cause recessive loss‐of‐function through impaired CPLANE2 interaction, resulting in multi‐organ ciliopathy, while LD1/LD3 variants may exert dominant‐negative effects on complex assembly, manifesting as more restricted neural tube defects. This model requires functional validation but provides a potential framework for predicting both inheritance patterns and disease severity based on variant location within *FUZ*.

In conclusion, this report provides additional evidence supporting *FUZ* as a causative gene for ciliopathy, suggesting that primary cilia dysfunction may contribute to the pathogenesis of AP window and Hirschsprung disease. Our findings indicate that altered FUZ‐CPLANE2 interactions may underlie the ciliopathy phenotype in this patient. Future functional analyses are expected to elucidate the mechanisms underlying *FUZ*‐mediated ciliogenesis and the pathogenesis of *FUZ*‐related ciliopathy.

## Author Contributions

Y.O. and R.I. designed the study, performed analyses, interpreted the results, and drafted the manuscript; Y.O. and S.K. contributed to laboratory work and data interpretation; Y.O., K.S., and R.I. were involved in clinical phenotyping; M.K. and R.I. supervised this study; all authors reviewed and discussed the manuscript during preparation and approved the final manuscript.

## Funding

This research was supported by AMED under Grant Number JP23ek0109760.

## Ethics Statement

The procedures employed were reviewed and approved by the central ethics committee at the University of Tokyo (#G3565).

## Consent

Informed consents were provided by the patient's parents to publish this report.

## Conflicts of Interest

The authors declare no conflicts of interest.

## Supporting information




**Table S1.** List of candidate variants.

**Table S2.** Summary of genotype and phenotype of the patients with FUZ‐related recessive ciliopathy.

## Data Availability

The data that supports the findings of this study are available in the Supporting Information of this article.
