# Supplementary material for: Phenotypic Expansion and Molecular Implications in Recessive FUZ ‐Related Ciliopathy
Source: Clin Genet. 2026 Apr 8;110(2):236–41. doi: 10.1111/cge.70170 (PMC13327168; doi:10.1111/cge.70170)
Supplement: Supplementary file 1 — Table S1. List of candidate variants. [file CGE-110-236-s002.docx]

**Table S1. List of candidate variants.**

We filtered variants based on the following criteria: (i) Allele frequency (AF) < 0.0001 (autosomal dominant/X-linked) or < 0.001 (autosomal recessive) in Japanese (ToMMo 60KJPN), (ii) deleterious in *in silico* pathogenicity assessment tools (SIFT, PolyPhen, CADD, and AlphaMissense (AM)). Among 15 candidate variants below, we screened for genes with previously reported phenotypes or functions consistent with our case.

| **Variant** | **Gene** | **Mutation** | **Zygosity** | **AF_60KJPN** | **AF_gnomAD(v4)** | **SIFT** | **PolyPhen** | **CADD** | **AM** |
| --- | --- | --- | --- | --- | --- | --- | --- | --- | --- |
| chr2_27033016_A/G | TMEM214 | p.M1V | Het | 0.000008 | 0.000007 | 0 | 0.768 | 24.1 | - |
| chr2_95182000_A/T | ZNF2 | p.H391L | Het | - | - | 0 | 0.992 | 24.3 | 0.9173 |
| chr2_119630902_GTAA/- | CFAP221 | Splice donor | Het | - | - | - | - | - | - |
| chr3_57577376_AAAAA  TAAGACCCTGGGG/- | ARF4 | Splice acceptor | Het | - | - | - | - | - | - |
| chr7_4755276_G/A | FOXK1 | p.V315M | Het | - | 0.000007 | 0 | - | 31.0 | 0.9923 |
| chr11_47642407_G/C | MTCH2 | p.P20R | Het | - | - | 0 | 0.802 | 29.8 | 0.9929 |
| chr11_47642411_G/A | MTCH2 | p.Q19* | Het | - | - | - | - | 38.0 | - |
| chr12_51110906_G/A | TFCP2 | p.P179S | Het | - | - | 0 | 0.667 | 26.0 | 0.7007 |
| chr14_44900923_G/C | C14orf28 | p.R163P | Het | 0.000017 | 0.000002 | 0 | 0.516 | 29.1 | 0.8198 |
| chr15_77133615_G/C | PEAK1 | p.P11156R | Het | 0.000017 | 0.000001 | 0 | 0.998 | 26.5 | 0.9186 |
| chr16_31362073_A/G | ITGAX | Splice acceptor | Het | 0.000017 | 0.000001 | - | - | 33.0 | - |
| chr17_41973736_C/T | CNP | p.R360* | Het | 0.000008 | 0.000009 | - | - | 37.0 | - |
| chr18_63641816_A/C | SERPINB4 | p.Y99D | Het | - | - | 0 | 1 | 24.7 | 0.3501 |
| chr19_12147780_A/G | ZNF625 | p.V9A | Het | 0.000008 | - | 0 | 0.993 | 22.8 | - |
| chr19_49809249_G/A | FUZ | p.R234W | Hom | 0.000117 | 0.000008 | 0 | 0.999 | 27.3 | 0.8440 |
