# Supplementary material for: Phenotypic Expansion and Molecular Implications in Recessive FUZ ‐Related Ciliopathy
Source: Clin Genet. 2026 Apr 8;110(2):236–41. doi: 10.1111/cge.70170 (PMC13327168; doi:10.1111/cge.70170)
Supplement: Supplementary file 2 — Table S2. Summary of genotype and phenotype of the patients with FUZ‐related recessive ciliopathy. [file CGE-110-236-s001.docx]

**Table S2. Summary of genotype and phenotype of the patients with FUZ-related recessive ciliopathy.**

|  | Case 1 | Case 2 | Case 3 | Case 4 | Case 5 | Our case |
| --- | --- | --- | --- | --- | --- | --- |
| **Patient** |  |  |  |  |  |  |
| Age | 2 years 9 months | Fetus (19 weeks of gestational age) | 6 months (monozygotic twin I) | 6 months (monozygotic twin II) | Fetus (24 weeks of gestational age) | 12 years |
| Sex | Female | NA | Female | Female | NA | Male |
| Ethnicity | Indian | Indian | NA | NA | African American | Japanese |
| Consanguinity | + | − | + | + | − | + |
| **Genotype** |  |  |  |  |  |  |
| Variant | p.Glu201Lys | p.Glu201Lys  p.Val209_Leu212del | p.Arg284Pro | p.Arg284Pro | c.98_111+9del | p.Arg234Trp |
| Zygosity | Homozygous | Compound heterozygous | Homozygous | Homozygous | Homozygous | Homozygous |
| **Birth information** |  |  |  |  |  |  |
| Gestational age | NA | − | 36 weeks 4 days | 36 weeks 4 days | − | 36 weeks 6 days |
| Birth weight | 2,800 g (-1.2SD) | − | 1,870 g | 2,125 g | − | 1,943 g (-2.3SD) |
| **Craniofacial malformations** | **NA** | **Yes** | **Yes** | **Yes** | **Yes** | **Yes** |
| Craniosynostosis | NA | NA | + (Metopic/coronal) | + (Metopic) | NA | + (Sagittal) |
| Cleft lip | − | + | − | − | + (Midline facial cleft) | − |
| Other facial features | Prominent forehead, medial flaring of the eyebrow, low-set ears, prominent antihelix | NA | Low hairline, prominent ears, narrow nose | Low hairline, prominent ears | NA | Micrognathia, high-arched palate |
| **Skeletal malformations** | **Yes** | **Yes** | **No** | **No** | **Yes** | **Yes** |
| Short ribs | − | − | − | − | + | − |
| Poly(syn)dactyly of fingers | − | + (Preaxial/postaxial) | − | − | + (Preaxial/postaxial) | + (Postaxial) |
| Poly(syn)dactyly of toes | + (Preaxial/postaxial) | + (Preaxial/postaxial) | − | − | + | + (Preaxial/postaxial) |
| **Cardiac malformations** | **Yes** | **Yes** | **No** | **No** | **Yes** | **Yes** |
| AVSD | + | + | − | − | + | + |
| Others | − | − | − | − | HLHS | DORV, AP window |
| **Cerebral malformations** | **NA** | **NA** | **Yes** | **Yes** | **Yes** | **Yes** |
| Hypoplasia of the callosal body | NA | NA | + | + | NA | + |
| Ventricular dilation | NA | NA | + (Lateral/third) | + (Lateral) | + (Third) | + (Lateral/fourth) |
| Others | NA | NA | − | − | NA | Hypoplasia of pons and middle cerebellar peduncle |
| **Neuro-developmental disorders** | **No** | **NA** | **Yes** | **Yes** | **NA** | **Yes** |
| Developmental delay | − | NA | + | + | NA | + |
| Others | − | NA | − | − | NA | Epilepsy, autism spectrum |
| **Other findings** | − | − | − | Premature puberty | Hypoplastic kidney | Hirschsprung disease |

AP window, aorto-pulmonary window; AVSD, atrioventricular septal defect; DORV, double outlet right ventricle; HLHS, hypoplastic left heart syndrome; NA, not available
